# Ammonia and hydrogen sulfide - new insights into gut microbiota and male infertility through meta-analysis

**DOI:** 10.3389/fcimb.2024.1449453

**Published:** 2025-01-06

**Authors:** Yanan Hao, Xin Du, Chang Cai, Yong Zhao, Yonglin Ren

**Affiliations:** ^1^ The Institute of Brain Science and Brain-inspired Research, Shandong First Medical University and Shandong Academy of Medical Sciences, Jinan, China; ^2^ College of Environmental and Life Sciences, Murdoch University, Perth, WA, Australia; ^3^ College of Life Sciences, Qingdao Agricultural University, Qingdao, China; ^4^ State Key Laboratory of Animal Nutrition, Institute of Animal Sciences, Chinese Academy of Agricultural Sciences, Beijing, China

**Keywords:** ammonia, hydrogen sulfide, gut microbiota, male infertility, internal gases

## Abstract

**Background:**

Ammonia (NH_3_) and hydrogen sulfide (H_2_S) are produced during digestion in the human gut, yet the impact of these internally generated gases on male reproduction have received limited attention in scientific research.

**Methods:**

We systematically reviewed 935 scientific publications, spanning from 1947 to 2023, focusing on external or internal NH_3_ and/or H_2_S, male infertility, and gut microbiota. Meta-analysis was conducted to evaluate the summary relative risk (RR) and 95% confidence intervals (CIs) of combined studies.

**Results:**

Our findings revealed that the internal NH_3_ and/or H_2_S were negatively related to the *Lactobacillus*, which is beneficial to male fertility, whereas NH_3_ and H_2_S were positively related to *Bacteroides*, which showed negative effects on male fertility. The meta-analysis comparing *Lactobacillus* and *Bacteroides* levels with NH_3_ showed statistically significant results (*p*<0.001).

**Conclusions:**

The meta-analysis is the first to confirm these facts and explored the potential existence of a gut microbiota-inner gases-male fertility axis in the human gut.

## 1 Introduction

Exposure to gaseous NH_3_ and H_2_S has detrimental effects on human health (Jankowski et al., 2014). These gases are emitted by various sources, including industry processes (Norizan et al., 2022), landfills (Jiang et al., 2021), livestock operations (Barrasa et al., 2012; Kim et al., 2008; Liu et al., 2014; Lu et al., 2020; Song et al., 2013; Wu et al., 2020; Zicari et al., 2013), and agriculture activities (Geiser et al., 2008; Li et al., 2023). NH_3_ and/or H_2_S not only adversely affect humans and livestock (Zicari et al., 2013) but also wild ecosystems (Dordevic´ et al., 2021; Geiser et al., 2008). As inorganic solutes, these gases exert multiple negative influences on the blood, breath, stools, and the gastrointestinal tract, including protein binding (Jankowski et al., 2014). Hydrogen sulfide (H_2_S) can cause eye damage even after brief exposure to low concentration (Lambert et al., 2006). Moreover, exposure to NH_3_ and H_2_S diminishes male reproductive ability by reducing sperm motility through AMPK/AKT-related pathways, and this damage may be heritable (Zhao et al., 2016; Zhang et al., 2018). Research has shown that external NH_3_ and H_2_S disrupt sperm parameters (e.g., sperm motility, sperm concentration) and the expression of spermatogenetic proteins via the energy metabolic pathway.

Apart from external sources, NH_3_ and H_2_S are also produced within the body. The generation of these gases in the body is related to the gut microbiota responsible for protein metabolization (Cai et al., 2022). Studies have revealed that the production of these gases can induce intestinal diseases (Wang et al., 2020; Yang et al., 2020), diabetes (Yang et al., 2020), obesity, central nervous system diseases, and cardiovascular diseases (Cai et al., 2022; Li et al., 2009). The gut microbiota performs crucial functions in the human body’s immunological, metabolic, structural, and neurological landscapes (Adak and Khan, 2019). Moreover, the gut microbiota plays a significant part in male fertility. Zhang et al. (2021a) and Zhang et al. (2021b) provided evidence that gut dysbiosis influences the fertility of male mice. The species profile of gut microbiota in infertile mice differs from that of fertile ones. The infertile group showed decreases in ‘beneficial’ bacteria and increases in ‘harmful’ bacteria. Grande et al. (2024) discovered a relationship between male tract microbiota and male infertility, highlighting interactions between the seminal and vaginal microbiota. According to Ashonibare et al. (2024) and Wang and Xie (2022), gut microbiota influences male reproductive function and behaviors in several ways, including alterations in ROS and sex hormones generation, and activation of cytokine accumulation and the immune system.

Therefore, a link between external NH_3_ and/or H_2_S and male fertility, as well as gut microbiota and male fertility, has been proven, but studies on the effects of internally generated NH_3_ and/or H_2_S gases are limited. NH_3_ and/or H_2_S is produced by the decomposition or metabolism of intestinal bacteria (Han et al., 2021; Wang et al., 2021), which is primarily influenced by daily diet (Kalantar-Zadeh et al., 2019), even the intestinal gas status, abdominal symptoms, and gastrointestinal disease state. Consequently, the results of existing studies are often inconsistent, and there needs to be more statistical power in the performed trials.

The rising cases of infertility worldwide have attracted more and more attention, and male factors are estimated to contribute to 30%-50% of the cases (Eisenberg et al., 2023). This study aims to evaluate the correlation between internally produced NH_3_ and/or H_2_S gases and gut microbiota related to male infertility through a systematic review and a meta-analytic approach. This study will provide novel insights into the production of internal body gases in relation to gut microbiota and male fertility.

## 2 Methods

In September 2022, a fuzzy inquiry was conducted on PubMed (www.ncbi.nlm.nih.gov/pubme) to gather literature on male infertility responses, gut microbiota, and NH_3_ and H_2_S. The search terms utilized included male infertility or sterility or reproductive problems or reproductive issues and gut microbiota, *Lactobacillus*, *Bacteroides*, and NH_3_ or ammonia / H_2_S or hydrogen sulfide. These terms were used both as free text and as subject headings, with the language restriction set to English. After the fuzzy inquiry searching, the published papers were narrowed down to 1947-2023, and 935 papers were recovered from PubMed data. The titles and abstracts of these papers were then screened, and papers with information relating to gut dysbiosis in male infertility or bacteria with gas emission were selected. After this screening process, 33 papers related to male infertility and gut microbiota, 141 papers on *Lactobacillus* and NH_3_ and/or H_2_S, and 36 papers on *Bacteroides* and NH_3_ and/or H_2_S were identified. Patients or the public were not involved in our research’s design, conduct, reporting, or dissemination plans.

Duplicate papers were removed, and the remainder assessed for the following criteria:

- Prospective, randomized, double-blind trials.
- A source of primary data, not a review.
- The sample size is given.
- Appropriate control and treatment groups.
- The aim of the study was closely related to our searched keywords.

**Figure 1:**
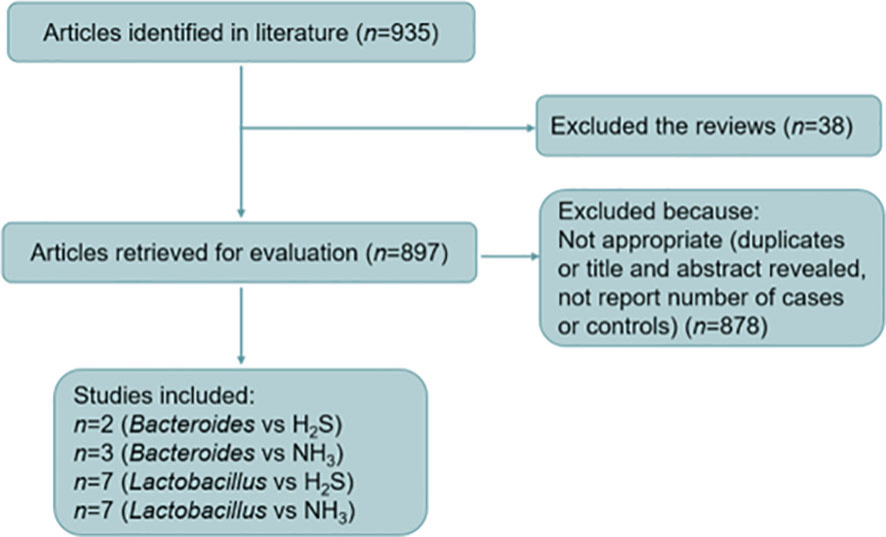
Flow diagram of study selection for the meta-analysis of bacteria vs. NH_3_ and H_2_S.

After screening, 19 research papers fulfilled all five criteria listed above. Meta-analyses were carried out in according to the published Preferred Reporting Items for Systematic Reviews and Meta-Analyses guidelines (Page et al., 2021). Descriptive statistical analysis was applied to summarize the total events from the 19 studies through RevMan v5.4 (Cochrane, London, UK) (**Figure 1**). Response to treatment was defined as the number of individuals with overall symptoms divided by the total number of individuals studied, reported as ratio (R) and 95% confidence interval (95% CI) and relative ratio (RR) and 95% CI for *Lactobacillus* and *Bacteroides*, respectively. Fixed- and random-effects models were used when *I* square ≤50% and *I* square >50%, respectively. The results were displayed as forest plots.

## 3 Results

### 3.1 Levels of *Lactobacillus* and *Bacteroides* changed in infertile males

**Figure 2:**
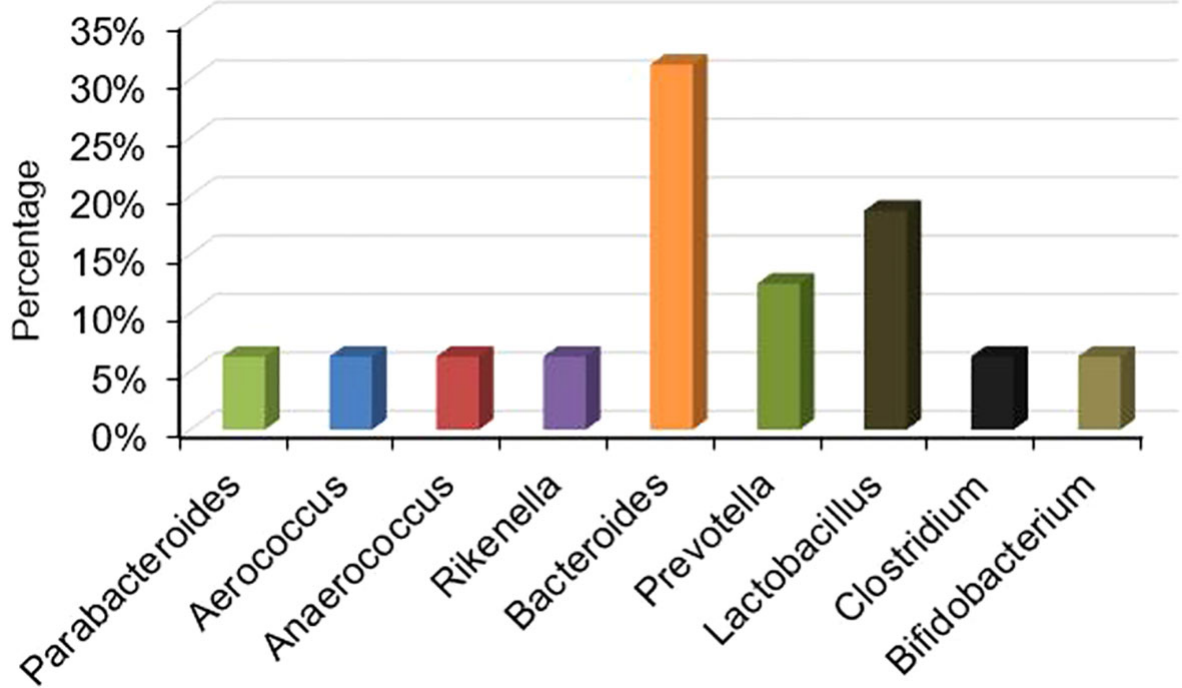
Percentage of different bacteria studied in the screened papers.

Gut dysbiosis has been associated with male infertility. In order to identify the critical variable bacteria in the infertile male, we summarized the results of altered bacterial profiles in sterile males (**Supplementary Table 1**). No matter how much the bacteria increased or decreased, we calculated the ratio of changed bacteria in 12 out of 33 studies. Notably, *Lactobacillus* and *Bacteroides* were the most frequently investigated bacteria in those research papers (**Figure 2**).

Since *Lactobacillus* and *Bacteroides* have been extensively studied in fertile and infertile males (19% and 31% respectively), we did a meta-analysis of the available literature on these two bacteria to further investigate their relationship with the production of NH_3_ and H_2_S in the gut.

### 3.2 Results of the meta-analysis of *Lactobacillus* vs. NH_3_ and/or H_2_S

We performed a meta-analysis to gain insight into the correlation between *Lactobacillus* species and NH_3_ and/or H_2_S in the gut. Seven studies related to *Lactobacillus* vs. H_2_S (**Supplementary Table 2**), and seven studies related to *Lactobacillus* vs. NH_3_ (**Supplementary Table 3**) were included in this analysis. The following is the formula of the random-effect model in this method:


$$Y_{i}=\mu +\xi +\epsilon_{i,}\epsilon_{i}=Y_{i}-\theta_{i}$$

Where: *Yi* is the observed effect in the study.

*μ* is the overall mean.

*θi* is the true mean of the studies.

*ξ* is the random variable.

**Figure 3:**
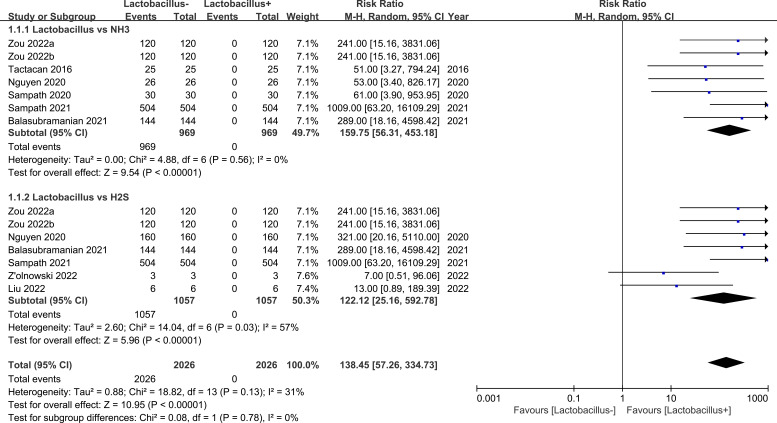
Meta-analysis of *Lactobacillus* vs. NH_3_ and H_2_S. Events means the number of the cases in which an event occurred. Total means the sample size for each group. + means rich, - means poor. M-H means effect amount.

The forest plot depicted in **Figure 3** reveals a statistically significant meta-analysis result (*p*<0.00001) comparing *Lactobacillus* to NH_3_, with a total effect indicated by a risk ratio (RR) of159.75 and a 95% confidence interval (CI) ranging from 56.31 to 453.18. According to the 1938 individuals in the analysis, it is suggested that there is less NH_3_ in the *Lactobacillus*-rich group. The *I* square showed that the included studies had a low heterogeneity (*I* square=0%). The forest plot result of the meta-analysis of 2114 individuals on *Lactobacillus* vs. H_2_S suggested less H_2_S in the *Lactobacillus*-rich group with RR=122.12 (95% CI 25.16-592.78). Although two studies showed no significant statistical analysis (Liu et al., 2022; Zolnowski et al., 2022), the total result was significant (*Chi* square=14.04, df=6, *I* square=57%, Z-test *p*<0.00001). Furthermore, the *p*-value of the Z-test was smaller than 0.00001, indicating that the analysis was highly significant. Based on the different Z-values of the subgroups (Z=9.54 (NH_3_)< 5.96 (H_2_S)), the NH_3_ has a higher probability of negative relationship with *Lactobacillus* than H_2_S. Even though there is slightly higher heterogeneity in *Lactobacillus* vs H_2_S, the total heterogeneity is less than 50% (*I* square=31%). These data indicated that *Lactobacillus* exhibits a significantly negative relationship with both NH_3_ and H_2_S in the gut.

This is the formula of Z-test in this study:

Z=(x-μ)/ ( σ/√n ) Where: *x* is the sample mean.

*μ* is the overall mean.

*σ* is the overall standard deviation.

*n* is the sample size.

Notes: Z value represents the different probability (Z=~-∞: 0%; Z=~0: 50%; Z=~+∞: 100%)

### 3.3 Results of the meta-analysis of *Bacteroides* vs. NH_3_ and H_2_S

We used a meta-analytic approach to understand if there was a correlation between *Bacteroides* levels and NH_3_ and H_2_S production in the gut. Two studies related to *Bacteroides* vs. H_2_S (143 individuals) (**Supplementary Table 4**) and three studies related to *Bacteroides* vs. NH_3_ (243 individuals) (**Supplementary Table 5**). The following is the formula of the fixed model in this method:


$$Y_{i}=\theta +\epsilon_{i}$$

Where: *Y_i_* is the observed effect in the study

*Θ* is the true effect in the study

*ϵi* is the difference between the true effect and the observed effect.

**Figure 4:**
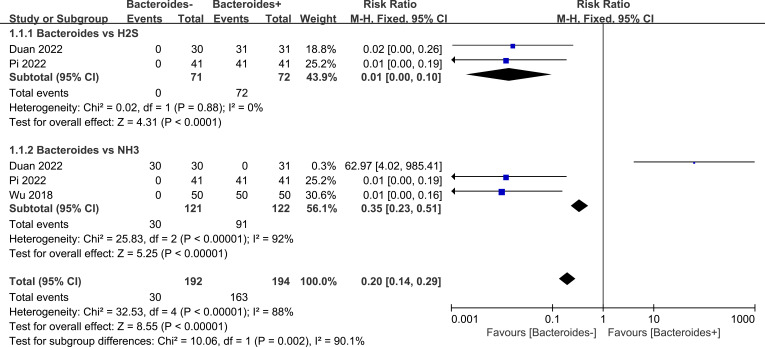
Meta-analysis of *Bacteroides* vs. NH_3_ and H_2_S. Events means the number of the cases in which an event occurred. Total means the sample size for each group. + means rich, - means poor. M-H means effect amount.

According to the forest plot (**Figure 4**), the meta-analysis of the *Bacteroides* vs. NH_3_ showed significant statistical results (*p*<0.001), the total RR=0.35 (95% CI 0.23-0.51). There is more NH_3_ in the *Bacteroides*-rich group. However, the *I* square indicated that the included studies had a higher heterogeneity (*I* square=92%). Furthermore, the forest plot result of meta-analysis on *Bacteroides* vs. H_2_S suggested that there are more H_2_S in the *Bacteroides*-rich group with RR=0.01 (95% CI 0.00-0.10). The *p*-value of the Z-test was smaller than 0.0001, which suggested that the analysis showed significant statistical results. Based on the different Z-values of the subgroup (Z=5.25 (NH_3_) > 4.31 (H_2_S)), the NH_3_ has a higher probability of a positive relationship with *Bacteroides* than the H_2_S. Despite the high heterogeneity among the included studies, the Z-test for the overall effect of *Bacteroides* vs. NH_3_ and *Bacteroides* vs. H_2_S demonstrated that *Bacteroides* showed a dramatic positive relationship with NH_3_ and H_2_S in the gut.

## 4 Discussion

This paper first proposed the potential axis linking gut microbiota, inner gases (NH_3_ or/and H_2_S produced by gut microbiota), and male fertility based on a comprehensive meta-analysis. Our investigation is the first meta-analysis study to investigate the NH_3_ and/or H_2_S in the gut involving male infertility.

The gut microbiota is a term that describes all microorganisms that inhabit the intestine (Chen et al., 2021; Pushpanathan et al., 2019). The gut microbiota engages in many important activities, such as maintaining physiological homeostasis and contributing to the physical performance of the host (Campaniello et al., 2022; Marttinen et al., 2020; Pushpanathan et al., 2019). Furthermore, gut flora communities play a pivotal role in diseases processes (Paoli et al., 2019). Gut dysbiosis shows adverse effects on the body (Chen et al., 2021), leading to increased permeability, endotoxemia, insulin resistance, and metabolic disorders (Pushpanathan et al., 2019). Besides that, the gut microbiota interacts with male fertility (Cai et al., 2022; Zhao et al., 2020; Zhang et al., 2021a and b) potentially contributing to testicular dysfunction by disrupting polyamine metabolism (Zhao et al., 2021). (). In this study, we synthesized the interaction between gut disorder and male infertility through a summary statistic. Among the variable bacteria in the included studies, those that changed noticeably were *Lactobacillus* (19%) and *Bacteroides* (31%). Interestingly, this finding aligns our previous study (Zhao et al., 2020; Zhang et al., 2021a and b), which reported significant alterations in *Lactobacillus* and *Bacteroides* in the gut of infertile male mice.

External sources of NH_3_ and H_2_S have been proven to induce male infertility (Zhang et al., 2018; Zhao et al., 2016). They diminish male fertility by reducing sperm motility through the AMPK/AKT pathway and could influence the offspring. It is known that the gut plays a crucial role in internal gas emissions (Kim et al., 2022). Our study first proposed the correlation between gut microbiota and internal NH_3_ and H_2_S by meta-analysis. As it was found that gut microbiota closely related to male fertility in our laboratory previous study by mice (Zhang et al., 2021a and b), this study further verified the interaction among gut microbiota, NH_3_ and H_2_S, and male fertility. The studies analyzed in this paper covered seven papers related to *Lactobacillus* and NH_3_ and seven papers related to *Lactobacillus* and H_2_S. The meta-analysis showed significant statistical data, indicating a negative association between *Lactobacillus* and these gases. Specifically, higher levels of *Lactobacillus* were associated with lower NH_3_ and H_2_S levels in the gut, which the outcomes are consistent with our previous work. Zhang et al. (2018) and Zhao et al. (2016) have demonstrated that NH_3_ and H_2_S adversely affect the fertility of male mice and boars. In humans, *Lactobacillus* sp. are the overwhelmingly dominant bacteria within reproductive tissues (Poole et al., 2023). Interestingly, *Lactobacillus* sp. have been found associated with improvements in semen parameters (Farahani et al., 2021). *Lactobacillus* could extend the male mating time and result in higher short-term offspring production in females (Morimoto et al., 2017). In addition, *Lactobacillus* could ameliorate the total and progressive motility and acrosome integrity (Mahiddine et al., 2022) and the testicular function (Çiftci and Tuna, 2021). *Lactobacillus* is not only beneficial for male fertility but also better for restraining the negative influence of *Pseudomonas* (Zhang et al., 2020). This study confirmed previous findings that showed a negative correlation between *Lactobacillus* and gut NH_3_ and H_2_S production further demonstrating the benefits of *Lactobacillus* on male fertility through mediating gas generation.

This study showed that the amount of *Bacteroides* is positively related to the concentration of NH_3_ and H_2_S. The heterogeneity of the meta-analysis of *Bacteroides* vs. NH_3_ is high because of the differences in the research analyzed in this study. Duan et al. (2022) showed data that was opposite to Pi et al. (2022) and Wu et al. (2018). Unlike the later studies, Duan et al. (2022) suggested more NH_3_ in the low *Bacteroides* group. However, the final Z-test of the published studies included in this analysis showed that increased levels of *Bacteroides* sp. are positively related to NH_3_. Combining the two subgroup results of meta-analysis, *Bacteroides* positively related to NH_3_ and H_2_S. Some investigations have proved the beneficial role of the *Bacteroides* in the gut (Singh, 2019). Microbiotas contribute substantially to the well-being of the host, including the immune response and nervous system (Strandwitz et al., 2019); however, different locations of the bacteria presented could also be pathogenic (Zafar and Saier, 2021). While *Bacteroides* species are part of the normal gastrointestinal microbiota they are also the most common anaerobic infective bacteria with an associated mortality of 19%. *Bacteroides* species have high resistance to antibiotics and contain antibiotic resistance mechanisms (Jasemi et al., 2021; Wexler, 2007). Quaglio et al. (2022) stated that *Bacteroides* species are associated with chronic tissue inflammation and the release of pro-inflammatory and carcinogenic mediators, upregulating the chance of developing colorectal cancer and irritable bowel syndrome. Ding et al. (2020) indicated that *Bacteroides* are related to metabolic endotoxemia, revealing a strong negative relationship with sperm motility and a positive correlation with blood endotoxin. Our study showed that *Bacteroides* has a positive relationship with the levels of NH_3_ and H_2_S. Based on our previous study on the external NH_3_ and H_2_S inducing male infertility (Zhang et al., 2018; Zhao et al., 2016), we hypothesize that *Bacteroides* might induce male sterility through increasing NH_3_ and H_2_S levels in the gut. However, the underlying mechanisms require further experimental verification. pH status has been shown to influence male fertility (Dai et al., 2024), and the intestinal pH closely correlates with the bacteria (Cotter and Hill, 2003). Herein, one hypothesis is that the internal NH_3_ and H_2_S produced by bacteria might affect male fertility by regulating the pH environment.

**Figure 5:**
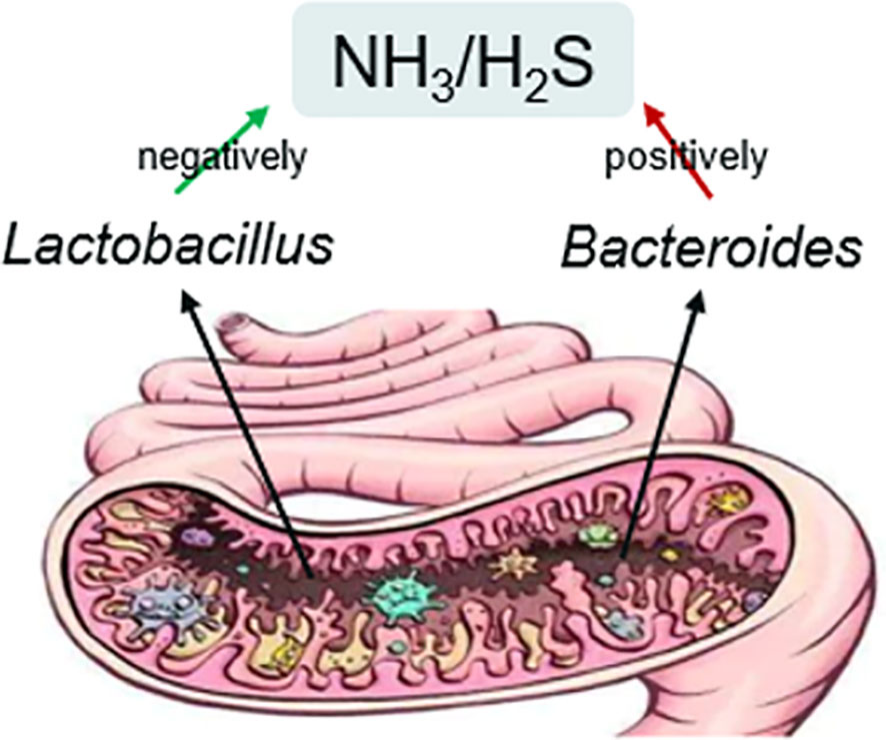
Relationship between gut microbiota and NH_3_ and H_2_S.

It is known that the gut environment is complex, and its composition is dynamic throughout the life of the host (Adak and Khan, 2019). The human microbiome plays a pivotal role in digestion, vitamin synthesis, and immune system training (Newcomb). Gut dysbiosis, characterized by an imbalance between the beneficial and harmful flora, has been linked to many diseases (Quaglio et al., 2022). Our investigation confirmed the interaction between gut microbiota and NH_3_ and H_2_S production in the gut based on meta-analysis, which gives fresh insights into gut flora-related diseases. Although some studies focused on gas emission after feeding the microbe (Payling et al., 2017) or the composition of human intestinal gases (Modesto et al., 2022), this study is first to propose an interaction between NH_3_ and H_2_S, and gut microbiota through a meta-analysis approach (**Figure 5**). Due to the limited number of studies included, we did not perform statistical tests to assess for publication bias. However, our findings further demonstrated the correlation among inner NH_3_ and H_2_S, the gut microbiota, and male infertility. This concept has been validated through meta-analysis for the first time. Oue meta-analysis indicated that lower NH_3_ and H_2_S levels are associated with a *Lactobacillus*-rich gut, whereas higher NH_3_ and H_2_S levels are linked to a *Bacteroides*-rich gut. Our meta-analysis concluded that *Lactobacillus* sp. might be beneficial bacteria for male fertility, while *Bacteroides* sp. may be harmful. This indicates that gut microbiota affects male fertility through small molecule chemicals, such as NH_3_ and H_2_S. These may further disrupt sperm motility by AMPK/AKT pathway to damage male fertility (Zhao et al., 2016).

Our study indicated a potential connection among animals ‘internal’ NH_3_ and/or H_2_S (produced in the gut by the microbiota), gut microbiota composition and male infertility via meta-analysis. This study provides a new perspective on the relationship between gut bacteria and male reproduction, highlighting the potential involvement of *Bacteroide* and *Lactobacillus* in male fertility via small molecule chemicals NH_3_ and H_2_S produced in the gut. Notably, our focus was on meta-analysis and did not encompass specific animal experiments. However, this new finding could lead to the development of novel methods or designs for future research and provide a novel insight for the future animal experiments. Indeed, this underscores the strengths and significance of conducting a meta-analysis. Satisfying data based on these would decrease the heterogeneity of the observation.

## Data Availability

The raw data supporting the conclusions of this article will be made available by the authors, without undue reservation.
