# Supplementary material for: Ammonia and hydrogen sulfide - new insights into gut microbiota and male infertility through meta-analysis
Source: Front Cell Infect Microbiol. 2025 Jan 6;14:1449453. doi: 10.3389/fcimb.2024.1449453 (PMC11743720; doi:10.3389/fcimb.2024.1449453)
Supplement: Supplementary file 1 [file Table1.docx]

**Table 1.** Summary of results of studied bacteria in infertile male.

| **References** | **Country** | **Model species** | **Changed bacteria** | | |
| --- | --- | --- | --- | --- | --- |
| Zhao *et al.*, 2021 | China | Mice | | *Parabacteroides* |  |
| Lundy *et al.*, 2021 | United States | Human | | *Prevotella* and anaerobes*,* rectal *Anaerococcus* |  |
| Zhu *et al.*, 2022 | China | Yak | | *Rikenellaceae, Bacteroides, Prevotellaceae UCG-004, Bacteroidales RF16,* and *Alloprevotella DgA-11* |  |
| Morimoto *et al.*, 2017 | United Kingdom | *Drosophila* | | *Lactobacillus* |  |
| Al-Asmakh *et al.*, 2014 | Sweden | Mice | | *Clostridium Tyrobutyricum* |  |
| Feng and Liu, 2022 | China | / | | *Lactobacillus, Bacteroides* |  |
| Mahiddine *et al.*, 2022 | South Korea | Dog | | *Firmicutes, Proteobacteria,* |  |
| Zhao *et al.*, 2020 | China | Mice | | *Lactobacillaceae, Bacteroidales, Proteobacteria* |  |
| Fabozzi *et al.*, 2022 | Italy | Human | | *Clostidium* |  |
| Zhang *et al.*, 2022 | China | Sheep | | *Ruminococcaceae NK4A214* |  |
| Ding *et al.*, 2019 | China | Mice | | *Bacteroides, Prevotella* |  |
| Zhang *et al.*, 2021b | China | Mice | | *Bacteroides, Bifidobacteria, Sphigomonadales* and *Camphlobacterales* |  |

**Table 2.** Summary of results of H_2_S changed with *Lactobacillus*.

| **Reference** | **Country** | **Model species^1^** | **H_2_S in *Lactobacillus-rich***  **Group^2^** |
| --- | --- | --- | --- |
| Zołnowski *et al.*, 2022 | Poland | / |  |
| Liu *et al.*, 2022 | China | Mice |  |
| Balasubramanian *et al.*, 2021 | Korea | Broilers |  |
| Sampath *et al.*, 2021 | Korea | Broilers |  |
| Zou *et al.*, 2022a | China | Broilers |  |
| Zou *et al.*, 2022b | China | Broilers |  |
| Nguyen and Kim, 2020 | China | Broilers |  |

1. Broilers refers to meat chickens.
2. The direction of change in H_2_S in *Lactobacillus*-rich group vs control group. The black arrow represents the change of H_2_S concentration.

**Table 3.** Summary of results of NH_3_ changed with *Lactobacillus*.

| **References** | **Country** | **Model species^1^** | **NH_3_ in *Lactobacillus-rich***  **Group^2^** |
| --- | --- | --- | --- |
| Sampath *et al.*, 2021 | Korea | Broilers |  |
| Tactacan *et al.*, 2016 | Korea | Weanling Pigs |  |
| Balasubramanian *et al.*, 2021 | Korea | Broilers |  |
| Sampath *et al.*, 2020 | Korea | Finishing Pigs |  |
| Zou *et al.*, 2022a | China | Broilers |  |
| Zou *et al.*, 2022b | China | Broilers |  |
| Nguyen and Kim, 2020 | China | Broilers |  |

1. Broilers refers to meat chickens.

2. The direction of change in NH_3_ in *Lactobacillus*-rich group vs control group. The black arrow represents the changes of NH_3_ concentration.

**Table 4.** Summary of results of H_2_S changed with *Bacteroides*.

| **References** | **Country** | **Model species** | **H_2_S in *Bacteroides*-rich group^a^** |
| --- | --- | --- | --- |
| Duan *et al.*, 2022 | China | Human |  |
| Pi *et al.*, 2022 | China | Human |  |

1. The direction of change in H_2_S in *Lactobacillus*-rich group vs control group. The black arrow represents the changes of H_2_S concentration.

**Table 5.** Summary of results of NH_3_ changed with *Bacteroides*.

| **References** | **Country** | **Model species** | **NH_3_ in *Bacteroides*-rich group^a^** |
| --- | --- | --- | --- |
| Duan *et al.*, 2022 | China | Human |  |
| Pi *et al.*, 2022 | China | Human |  |
| Wu *et al.*, 2018 | China | Rabbit |  |

1. The direction of change in NH_3_ in *Lactobacillus*-rich group vs control group. The black arrow represents the changes of NH_3_ concentration.

**References:**

Al-Asmakh, M., J. B. Stukenborg, A. Reda, F. Anuar, M. L. Strand, L. Hedin, S. Pettersson, and O. So¨der. 2014. “The Gut Microbiota and Developmental Programming of the Testis in Mice.” *Plos One* 9, no.8 (August): e103809.

Balasubramanian, B., S. Shanmugam, S. Park, N. Recharla, J. S. Koo, I. Andretta, and I. H. Kim. 2021. “Supplemental Impact of Marine Red Seaweed (Halymenia palmata) on the Growth Performance, Total Tract Nutrient Digestibility, Blood Proﬁles, Intestine Histomorphology, Meat Quality, Fecal Gas Emission, and Microbial Counts in Broilers.” *Animals* 11, no.5 (April): 1244.

[Ding](https://pubmed.ncbi.nlm.nih.gov/?term=Ding+N&cauthor_id=31900292), N., [X. Zhang](https://pubmed.ncbi.nlm.nih.gov/?term=Zhang+X&cauthor_id=31900292), [X. D. Zhang](https://pubmed.ncbi.nlm.nih.gov/?term=Zhang+XD&cauthor_id=31900292), [J. Jing](https://pubmed.ncbi.nlm.nih.gov/?term=Jing+J&cauthor_id=31900292), [S. S. Liu](https://pubmed.ncbi.nlm.nih.gov/?term=Liu+SS&cauthor_id=31900292), [Y. P. Mu](https://pubmed.ncbi.nlm.nih.gov/?term=Mu+YP&cauthor_id=31900292), [L. L. Peng](https://pubmed.ncbi.nlm.nih.gov/?term=Peng+LL&cauthor_id=31900292), [Y. J. Yan](https://pubmed.ncbi.nlm.nih.gov/?term=Yan+YJ&cauthor_id=31900292), [G. M. Xiao](https://pubmed.ncbi.nlm.nih.gov/?term=Xiao+GM&cauthor_id=31900292), [X. Y. Bi](https://pubmed.ncbi.nlm.nih.gov/?term=Bi+XY&cauthor_id=31900292), [H. Chen](https://pubmed.ncbi.nlm.nih.gov/?term=Chen+H&cauthor_id=31900292), [F. H. Li](https://pubmed.ncbi.nlm.nih.gov/?term=Li+FH&cauthor_id=31900292), [B. Yao](https://pubmed.ncbi.nlm.nih.gov/?term=Yao+B&cauthor_id=31900292), and [A. Z. Zhao](https://pubmed.ncbi.nlm.nih.gov/?term=Zhao+AZ&cauthor_id=31900292). 2020. “Impairment of spermatogenesis and sperm motility by the high-fat diet-induced dysbiosis of gut microbes.” *Gut* 69, no.9 (September): 1608-1619.

Duan, Y. F., X. L. Wu, Y. N. Yang, L. Q. Gu, L. Liu, Y. F. Yang, J. Z. Zhou, C. M. Wu, and F. Jin. 2022. “Marked shifts in gut microbial structure and neurotransmitter metabolism in fresh inmates revealed a close link between gut microbiota and mental health: A case-controlled study.” *International Journal of Clinical and Health Psychology* 22, no.3 (September-December): 100323.

[Fabozzi](https://pubmed.ncbi.nlm.nih.gov/?term=Fabozzi+G&cauthor_id=36359730), G., [P. Rebuzzini](https://pubmed.ncbi.nlm.nih.gov/?term=Rebuzzini+P&cauthor_id=36359730), [D. Cimadomo](https://pubmed.ncbi.nlm.nih.gov/?term=Cimadomo+D&cauthor_id=36359730), [M. Allori](https://pubmed.ncbi.nlm.nih.gov/?term=Allori+M&cauthor_id=36359730), [M. Franzago](https://pubmed.ncbi.nlm.nih.gov/?term=Franzago+M&cauthor_id=36359730), [L. Stuppia](https://pubmed.ncbi.nlm.nih.gov/?term=Stuppia+L&cauthor_id=36359730), [S. Garagna](https://pubmed.ncbi.nlm.nih.gov/?term=Garagna+S&cauthor_id=36359730), [F. M. Ubaldi](https://pubmed.ncbi.nlm.nih.gov/?term=Ubaldi+FM&cauthor_id=36359730), [M. Zuccotti](https://pubmed.ncbi.nlm.nih.gov/?term=Zuccotti+M&cauthor_id=36359730), and [L. Rienzi](https://pubmed.ncbi.nlm.nih.gov/?term=Rienzi+L&cauthor_id=36359730). 2022. “Endocrine-Disrupting Chemicals, Gut Microbiota, and Human (In)Fertility-It Is Time to Consider the Triad.” *Cells* 11, no.21 (October): 3335.

Feng, T., and Y. Liu. 2022. “Microorganisms in the reproductive system and probiotic's regulatory effects on reproductive health.” *Computational and Structural Biotechnology Journal* 20, (March): 1541-1553.

Liu, J., W. Zhao, Z. W. Gao, N. Liu, W. H. Zhang, and H. Ling. 2022. “Effects of Exogenous Hydrogen Sulﬁde on Diabetic Metabolic Disorders in db/db Mice Are Associated with Gut Bacterial and Fungal Microbiota.” *Frontiers in Cellular and Infection Microbiology* 12, (March): 801331.

Lundy, S. D., N. Sangwan, N. V. Parekh, M. K. P. Selvam, S. Gupta, P. McCaffrey, K. Bessoff, A. Valad, A. Agarval, E. S. Sabanegh, S. C. Vij, and C. Eng. 2021. “Functional and Taxonomic Dysbiosis of the Gut, Urine, and Semen Microbiomes in Male Infertility.” *European Urology* 79, (January): 826-836.

Mahiddine, F. Y., I. You, H. Park, and M. J. Kim. 2022. “Commensal Lactobacilli Enhance Sperm Qualitative Parameters in Dogs.” *Frontiers in Veterinary Science* 9, (June): 888023.

Morimoto, J., S. J. Simpson, and F. Ponton. 2017. “Direct and trans-generational effects of male and female gut microbiota in Drosophila melanogaster.” *Biology Letters* 13, no.7 (July): 20160966.

Nguyen, D. H., and I. H. Kim. 2020. “Protected Organic Acids Improved Growth Performance, Nutrient Digestibility, and Decreased Gas Emission in Broilers.” *Animals* 10, no.3 (March): 416.

Pi, X. E., Z. C. Yu, X. X. Yang, Z. Du, and W. Liu. 2022. “Effects of Zymosan on Short-Chain Fatty Acid and Gas Production in in vitro Fermentation Models of the Human Intestinal Microbiota.” *Frontiers in Nutrition* 9, (July): 921137.

Sampath, V., S. Shanmugam, J. H. Park, and I. H. Kim. 2020. “The Eﬀect of Black Pepper (Piperine) Extract Supplementation on Growth Performance, Nutrient Digestibility, Fecal Microbial, Fecal Gas Emission, and Meat Quality of Finishing Pigs.” *Animals* 10, no.11 (October): 1965.

Sampath, V., D. H. Baek, S. Shanmugam, and I. H. Kim. 2021. “Dietary Inclusion of Blood Plasma with Yeast (Saccharomyces cerevisiae) Supplementation Enhanced the Growth Performance, Nutrient Digestibility, Lactobacillus Count, and Reduced Gas Emissions in Weaning Pigs.” *Animals* 11, no.3 (March): 759.

Tactacan, G. B., S. Y. Cho, J. H. Cho, and I. H. Kim. 2016. “Performance Responses, Nutrient Digestibility, Blood Characteristics, and Measures of Gastrointestinal Health in Weanling Pigs Fed Protease Enzyme.” *Asian-Australasian Journal of Animal Sciences* 29, no.7 (July): 998-1003.

Wu, Z. Y., H. L. Zhou, F. C. Li, N. B. Zhang, and Y. L. Zhu. 2018. “Effect of dietary fiber levels on bacterial composition with age in the cecum of meat rabbits.” *Microbiology Open* 8, no.5 (May): e00708.

Zhang, T., P. Sun, Q. Geng, H. T. Fan, Y. T. Gong, Y. T. Hu, L. Y. Shan, Y. C. Sun, W. Shen, and Y. Zhou. 2022. “Disrupted spermatogenesis in a metabolic syndrome model: the role of vitamin A metabolism in the gut- testis axis.” *Gut* 71, (January):78-87.

Zhang, C., B. H. Xiong, L. Chen, W. Ge, S. Yin, Y. N. Feng, Z. Y. Sun, Q. Y. Sun, Y. Zhao, W. Shen, and H. F. Zhang. 2021b. “Rescue of male fertility following fecal microbiota transplantation from alginate oligosaccharide dosed mice.” *Gut* 70, no.11 (December): 1608-1619

Zhao, [Q.](https://pubmed.ncbi.nlm.nih.gov/?term=Zhao+Q&cauthor_id=34758869), [J. F. Huang](https://pubmed.ncbi.nlm.nih.gov/?term=Huang+JF&cauthor_id=34758869), [Y. Cheng](https://pubmed.ncbi.nlm.nih.gov/?term=Cheng+Y&cauthor_id=34758869), [M. Y. Dai](https://pubmed.ncbi.nlm.nih.gov/?term=Dai+MY&cauthor_id=34758869), [W. F. Zhu](https://pubmed.ncbi.nlm.nih.gov/?term=Zhu+WF&cauthor_id=34758869), [X. W. Yang](https://pubmed.ncbi.nlm.nih.gov/?term=Yang+XW&cauthor_id=34758869), [F. J. Gonzalez](https://pubmed.ncbi.nlm.nih.gov/?term=Gonzalez+FJ&cauthor_id=34758869), and [F. Li](https://pubmed.ncbi.nlm.nih.gov/?term=Li+F&cauthor_id=34758869). 2021. “Polyamine metabolism links gut microbiota and testicular dysfunction.” *Microbiome* 9, no.1 (November): 224.

Zhu, Y. B., X. Li, L. S. Zhaxi, S. L. Zhaxi, C. Yang, G. M. Sun, C. D. Yangji, and B. S. Wangdui. 2022. “House feeding pattern increased male yak fertility by improving gut microbiota and serum metabolites.” *Frontiers in Veterinary Science* 9, (September): 989908.

[Zhao](https://pubmed.ncbi.nlm.nih.gov/?term=Zhao+Y&cauthor_id=32194870), Y., [P. F. Zhang](https://pubmed.ncbi.nlm.nih.gov/?term=Zhang+P&cauthor_id=32194870), [W. Ge](https://pubmed.ncbi.nlm.nih.gov/?term=Ge+W&cauthor_id=32194870), [Y. N. Feng](https://pubmed.ncbi.nlm.nih.gov/?term=Feng+Y&cauthor_id=32194870), [L. Li](https://pubmed.ncbi.nlm.nih.gov/?term=Li+L&cauthor_id=32194870), [Z. Y. Sun](https://pubmed.ncbi.nlm.nih.gov/?term=Sun+Z&cauthor_id=32194870), [H. F. Zhang](https://pubmed.ncbi.nlm.nih.gov/?term=Zhang+H&cauthor_id=32194870), and [W. Shen](https://pubmed.ncbi.nlm.nih.gov/?term=Shen+W&cauthor_id=32194870). 2020. “Alginate oligosaccharides improve germ cell development and testicular microenvironment to rescue busulfan disrupted spermatogenesis.” *Theranostics* 10, no.7 (February): 3308-3324.

Z˙ ołnowski, C., T. Bakuła, E. Rolka, and A. Klasa. 2022. “Effect of Mineral–Microbial Deodorizing Preparation on the Value of Poultry Manure as Soil Amendment.” *International Journal of Environmental Research and Public Health* 19, no.24 (December): 16639.

Zou, Q. Q., W. S. Meng, T. L. Wang, X. Liu, and D. S. Li. 2022a. “Effect of Multi-strain Probiotics on the Performance of AA+ Male Broilers.” *Frontiers in Veterinary Science* 9, (December): 1098807.

Zou, Q. Q., X. Y. Fan, Y. H. Xu, T. L. Wang, and D. S. Li. 2022b. “Effects of dietary supplementation probiotic complex on growth performance, blood parameters, fecal harmful gas, and fecal microbiota in AA+ male broilers.” *Frontiers in Microbiology* 13, (December): 1088179.
